# Joint Use of e.Photoexpression^©^ and Photonarration: What Methodological Added Value?

**DOI:** 10.3389/fpubh.2021.691587

**Published:** 2021-09-13

**Authors:** Deyra Maéliane, Gay Chloé, Gerbaud Laurent, Berland Pauline, Pizon Frank

**Affiliations:** ^1^University Clermont Auvergne, CNRS, SIGMA Clermont, Institut Pascal, Clermont-Ferrand, France; ^2^Université Clermont Auvergne, CHU, CNRS, SIGMA Clermont, Institut Pascal, Clermont-Ferrand, France

**Keywords:** complementarity, methodological added value, e.Photoexpression^©^, photonarration, cancer and health perceptions, children

## Abstract

**Objectives:** The objective is to describe the informative value and the added value of a qualitative multiphase methodology in order to investigate the conceptions of children aged 6–11 on the determinants of health and cancer.

**Method:** This article provides an analysis of the “Determ'Ados” research protocol, a qualitative study in human and social sciences, carried out with children aged 6–11 years. This protocol, organized in three phases, addresses in the first global health with the tool “e.Photoexpression^©^,” in the second questions and knowledge around the topic of cancer with the tool “QC” and in the third cancer again with the “Photonarration” tool. The methodology of this innovative, open and exploratory research protocol aims to collect data relating to the experiences, declared practices and knowledge specific to each child who express themselves through photography and storytelling.

**Results:** The analysis of the Déterm'Ados methodology reveals a density and richness of results among all the children interviewed, even among the youngest: 1,498 productions (4 productions per child) were made by 381 children resulting in a wealth of data available thanks to the multiphase protocol. This massive qualitative survey brings complementarity as the collection phases progress and guarantees continuity in the discourse of each child which allows them to deepen their conceptions and to know how they create or not meaning between the determinants of health and cancer.

**Perspectives:** The density and quality of the proposals collected from the children reinforce the validity and rigor of the Determ'Ados methodology. Multiphase is the innovative aspect of the tools used. The e.Photoexpression^©^ and the Photonarration are complementary and inseparable to bring out concepts on health and cancer. These research results, transferable into interventions and current practices, present prevention officers to act more effectively, closer to the conceptions and needs of children.

## Introduction

Public health has particularly evolved over the years toward a more global approach, integrating influential factors such as the social, economic and ecological dimensions of health (1). Global health is therefore not limited to identifying infectious and functional causes in pathological cases but also to highlighting environmental, psychosocial and socioeconomic reasons creating the context of social inequalities in health (2). Until the middle of the 20th century, health status depended mainly on the spread of infectious diseases such as cholera or tuberculosis. Today, it is mainly chronic diseases, including cancer, that affect health, diseases that are strongly correlated with living conditions and the environment. To promote health and reduce the risk of developing cancer, it is necessary to act on the determinants of health, which are “the personal, social, economic and environmental factors that determine the health status of individuals or populations” (3). It can therefore be stated, except for certain specific types of cancer, that the determinants of cancer are not diametrically opposed to those of health. Indeed, the increase in cancers and the general health status of the population both depend on several similar factors: population growth, aging and changes in the prevalence of certain causes of pathologies associated with social and economic conditions, poverty and infections (4), individual behaviors but also environmental determinants (5). The discussion here tends to confirm that there is a conceptual mesh between the notions of cancer determinants and health determinants, which ultimately resemble each other and come together.

Moreover, these determinants can be individual or collective, innate or acquired and interact with each other (6). The combination of their effects creates living conditions that affect overall health in specific ways (7). Differences in place of birth and education do not provide equal life chances. Health and disease follow a social gradient: the lower the socioeconomic position, the higher the risk of poor health (8). The unequal distribution of power, income, goods, and services, both globally and nationally, partly explains the significant disparities and resulting inequity in people's daily lives. This inequality affects their access to health care and education, their working and leisure conditions, their homes, their communities, their cities, and hinders their opportunities to lead fulfilling lives (9, 10).

Thus, there is ample evidence of the influence of the determinants of health (11). Today, the question is to understand how these determinants act so that we perceive their importance and thus know how to intervene more effectively in prevention (12). To understand the perceptions of the determinants of health that diffuse in our societies, research has shown the importance of investigating conceptions of health (13, 14). The term “conception of health” defines here what allows the individual to construct himself in both individual and collective dimensions. Conceptions include what allows the individual to characterize his or her health and what determines it in a biopsychosocial perspective (15).

Investigating conceptions in health remains a complex process and a real challenge for research even as the contexts of life are constantly evolving. As part of a research protocol, the combination of several survey methods seems to promote a better understanding of the phenomenon studied (16). This type of added value in a multiphase survey process also seems to be enriched by the added value of a mixed analysis (17). Multiple approaches would improve the research process and the argument by comparing the results from one method to another (18). They would make it possible to generalize preliminary or exploratory results, to confirm them and to discover new ones. Triangling the data collection would thus increase the validity of the study, fill the gaps in each method and develop a more complete and exhaustive perspective (19, 20). This type of added value in a multiphase survey process also seems to be enriched by the added value of a mixed analysis (16). Quantitative analysis offers researchers, just like qualitative analysis, tools to support them in their reasoning, in their empirical approach, in their research and in their analysis of survey data but it remains insufficient. It is important to have a fine qualitative analysis that allows to describe a subject rather than to measure it. The qualitative and the quantitative approach are therefore complementary (16, 19). They interact and enrich each other throughout the research process (21). This added value is crucial for this study because we are demonstrating that trying to describe conceptions of health or cancer is complex.

However, we have shown in a review of the literature (22) that few international publications use a qualitative or mixed collection methodology, centered on children's conceptions of health and cancer, and this despite a high and diversified level of knowledge and representations about health concepts (23, 24). The studies choose multiple methodological approaches, which are neither multiphase nor standardized and therefore not very reproducible. The most discussed health topics are mainly education in emotional and sexual life and the prevention of addictions and/or consumption of psychotropic drugs. Only three articles address the topic of cancer (25–27) while children and adolescents declare that they wish to receive more information on the subject (25). Finally, the use of a mixed analysis has rarely been mobilized. In this sparse bibliographical context, we wonder about the interest and relevance of invoking several survey methods coupled with a mixed analysis. The objective of this study, which is part of the “Determ'Ados” research project funded by the National Cancer League over 3 years (2019–2022), is therefore to describe the informative value and the added value of the methodological multiphase in understanding the conceptions of children aged 6–11, attending primary school, on the determinants of health and cancer.

## Presentation of the Determ'Ados Protocol

### Determ'Ados Data Collection Methodology

This is a qualitative and comprehensive study of Human and Social Sciences carried out with children enrolled in four French primary schools in the Auvergne-Rhône-Alpes region, Académie de Clermont-Ferrand. These establishments in the departments of Allier and Puy-de-Dôme have different contexts: rural or urban, Priority Education Network or not, small or large school. These sociodemographic data will be the subject of future publications, as the results presented in this article are sufficiently dense. All age groups were interviewed: 6–7 years old (preparatory class CP), 7–8 years old (elementary class CE1), 8–9 years old (second-year elementary CE2), 9–10 years old (middle class CM1), 10–11 years old (second-year middle class CM2); 18 classes in total. The data were collected from a multiphase qualitative study protocol that revolves around two tools: the e.Photoexpression^©^ and the Photonarration. This multiphase, open and exploratory method, mobilizing the use of photographs, is a support for speech and makes it possible to collect the experiences of each child in order to better understand the way in which he or she considers the determinants of health and cancer. These image mediation methods play an obvious and essential ethical role by ensuring that the subject matter is distanced from the child. He or she does not speak about his or her personal situation but only about the object of this study: the determinants of health and cancer. In addition, each child was assigned a code that facilitated, for each phase of the survey, perfect traceability of what is expressed. The absence of personal data also guarantees the impossibility of identification of children and anonymity. This research in Human and Social Sciences does not fall under “research involving the human person” (RIPH). An ethics certificate outside the Jardé law was issued to the Determ'Ados protocol by the South-East VI “Committee for the Protection of Persons” in order to publish the results.

The intervention protocol for this study was designed based on data from the literature, international recommendations, hypotheses derived from the professional experience of researchers and the methodology used in previous studies on health and addictions (28). The first phase with the “e.Photoexpression^©^” tool consists of asking the children to choose two images from 40 photographs that best answer the question: “Choose an image that you think represents good health” and “Choose an image which you think represents bad health.” Once the selection has been made, everyone explains in writing the reasons for their choice. The objective of e.Photoexpression^©^ is to have children identify the determinants having a favorable or unfavorable influence on health. This tool, validated and referenced PIPSA,^1^ registered INPI^2^ is protected by copyright (29). To build this body of photographs, several criteria were met. First, an aesthetic criterion including sharpness and framing. Then a criterion of meaning which corresponds to the meanings put through the image. The image must be open to offer a diversity of reading. Finally, a criterion of homogeneity which results in a fairly wide spectrum of photographs allowing everyone to express themselves.

The second phase with the “Photonarration” tool consists of choosing images without limit of number from a corpus of magazines (leisure, daily life, decoration, various specialized magazines, etc.), to cut them, assemble them and paste them on an A3 sheet to answer to the following instruction: “create an assembly of images from the magazines provided that shows the causes and protective factors for cancer.” Each production made is associated with a text on the back of the sheet answering the following questions: “What in the pictures you have chosen represents for you what causes cancer” and “What in the photographs you have chosen represents what helps prevent cancer.”

The objective of Photonarration is to bring out the children's conception systems in order to identify the argumentative frameworks they construct and the alternatives they consider, perceived as favorable or unfavorable for health and in relation to cancer.

The involvement of children in research is essential to guarantee their right to participate in debate on areas that affect them (International Convention on the Rights of the Child)^3^ and to improve the value and validity of the results. Research involving children is essential to understanding their conceptions. It ensures that their experiences and perspectives are properly identified, providing accurate and specific information, respecting their word. This is why this study is conducted with integrity and with respect for their dignity, opinions and culture. Respectful involvement of the child requires recognizing their status and developmental capacities. Children were treated equally and without exclusions. For this, the data collection conditions have been adapted. The little ones (CP and CE1) or all those for whom writing remains fragile were supported by researchers or teachers in the form of dictation to adults. This approach thus offers everyone the same opportunity to express themselves. No influence or help is provided from the adult who transcribes only the child's words. Finally, ethical research must be able to benefit children both as subjects in their own right and as a social group. The aim of this study is to collect data, analyze it and advance research, but it is also part of a prevention approach that provides an essential ethical framework in a field of research involving children.

### Presentation of the Determ'Ados Analysis Methodology

This qualitative survey made it possible to stabilize a mixed, qualitative and quantitative analysis protocol, necessary for in-depth production of results. The data collected through e.Photoexpression^©^ and Photonarration were entered in Excel^©^, respecting the children's writings identically. They were then analyzed quantitatively and qualitatively by the researchers as well as using the Gephi 9.2^©^ software.

To identify references to determinants of health and cancer in the children's discourse, content analysis was performed using the bottom-up/top-down categorization method and the “heap” procedure (30).

In order to accurately categorize the verbatims, we created our indexing grid under 4 different levels: domains, categories, themes and sub themes. The domains correspond to headings of general and global elements that group and structure the categories, themes and sub-themes. Each domain encompasses a dominant idea related to a determinant of health. A category corresponds to a set of common components that have common characteristics, “of the same nature.” It adds a descriptive element to its domain. A theme is a descriptive idea that is the main subject of the words corresponding to the category and domain to which it is affiliated. It adds a descriptive element to the category it belongs to. A sub-theme is a more detailed and specific idea than the theme. It adds a descriptive element to the theme it belongs to.

This classification produces a funnel effect with a granularity of ideas that becomes more refined as we go down from level to level. From one child to another, the cursor varies. We place the words of a child who remains evasive in the domain level. On the contrary, those who are more precise and detailed are classified in the sub-theme level. In order to verify the accuracy of the nesting of the different levels, the categories were stabilized on the basis of the children's discourse and a retrocontrol was carried out with the model of the determinants of health, the theoretical basis of this study (31).

In addition, the data were triple encoded to limit indexing bias. A first encoding, carried out by each researcher in a bottom-up/ top-down manner, allowed to verify that the domains were working, a second one to validate and enrich the first one, and finally a third one to confirm the results of the first two and to correct possible errors. To avoid over-interpretation of the collected verbatims, in case of doubt to index them, the contentious data were discarded after a thorough examination. The triple encoding of the qualitative analyses, as well as those carried out with Gephi 9.2^©^ software, were translated into quantitative data allowing for statistical analyses, enriched by regular feedback on the verbatims brought to light during the qualitative analysis. These exchanges between the qualitative and the quantitative analyses avoid over-interpretation, thus guaranteeing the validity of the results.

To complete the qualitative analysis, the data visualization software Gephi 9.2^©^ was used to explore the most central elements of a network, the best connected but also the most distant. It is a complementary tool to statistical analysis and is positioned as a means of pictorial representation of data that enhances children's trends by highlighting the determinants of health that they perceive. In order to establish networks between the data and to identify links, if any, a careful database was created so that the Gephi 9.2^©^ software could make an accurate reading and analysis. In order for the software to be able to graphically represent the organization of the network formed by our verbatims, it needs two pieces of information: a list of the actors composing the network to generate the nodes and a list of the relations between these actors to generate the edges. In our study, the actors correspond to the names of the items we have identified and their indexing categories. These criteria allow the Gephi 9.2^©^ software to represent in a pictorial way the links made by children between the determinants of health.

The methodology of survey and analysis allow to put forward very rich results.

## Putting the Results of the Overall Analysis of the Phases of the Determ'Ados Protocol Into Perspective

With our objective of highlighting the added value of the Determ'Ados protocol in this article, four major elements appear: (1) a density of results, (2) a variety of results from an early age, (3) discourse tendencies and (4) discourse complementarity.

### Density of Results

The first added value is the high density of the data collected, ensured by the multi-phase, which brings rich results for the entire age group 6–11 years (from CP to CM2):

- CP: 95 children of 6–7 years old
- CE1: 82 children of 7–8 years old
- CE2: 91 children of 8–9 years old
- CM1: 58 children of 9–10 years old
- CM2: 55 children of 9–0 years old.

1,498 productions, 4 productions per child (2 productions e.Photoexpression^©^: good/bad health and 2 productions Photonarration: prevent/causes cancer) were collected from 381 children (Table 1). During the first phase of the survey, 381 children, from CP to CM2, participated in the e.Photoexpression^©^ and realized 762 productions. The second phase included 368 children and collected 736 Photonarration productions. The difference in the total number of participants between the two survey phases is explained by absences due to benign childhood illnesses or due to transport difficulties related to snow.

**Table 1:** Sociodemographic data.

**School**	**Context**		**Classrooms**		**Number of children**	
	**Localization**	**Priority education network**	**Total number**	**Number visited**	**Total**	**Classrooms levels visited** **CP 6–7 years old** **CE1 7–8 years old** **CE2 8–9 years old** **CM1 9–10 years old** **CM2 10–11years old**
1	Countryside	No	7	4	184	24 CP 25 CE1 29 CE2 25 CP/CE1
2	Half city Half countryside	No	9	4	227	22 CP 21 CE1 27 CE2 26 CM1/CM2
3	City	Yes	15	5	280	15 CP 20 CE1 20 CE2 23 CM1/CM2 23 CM1/CM2
4	City	No	6	5	128	21 CP 20 CE1 24 CE2 24 CM1 25 CM2

### Variety of Results

**Figure 1:**
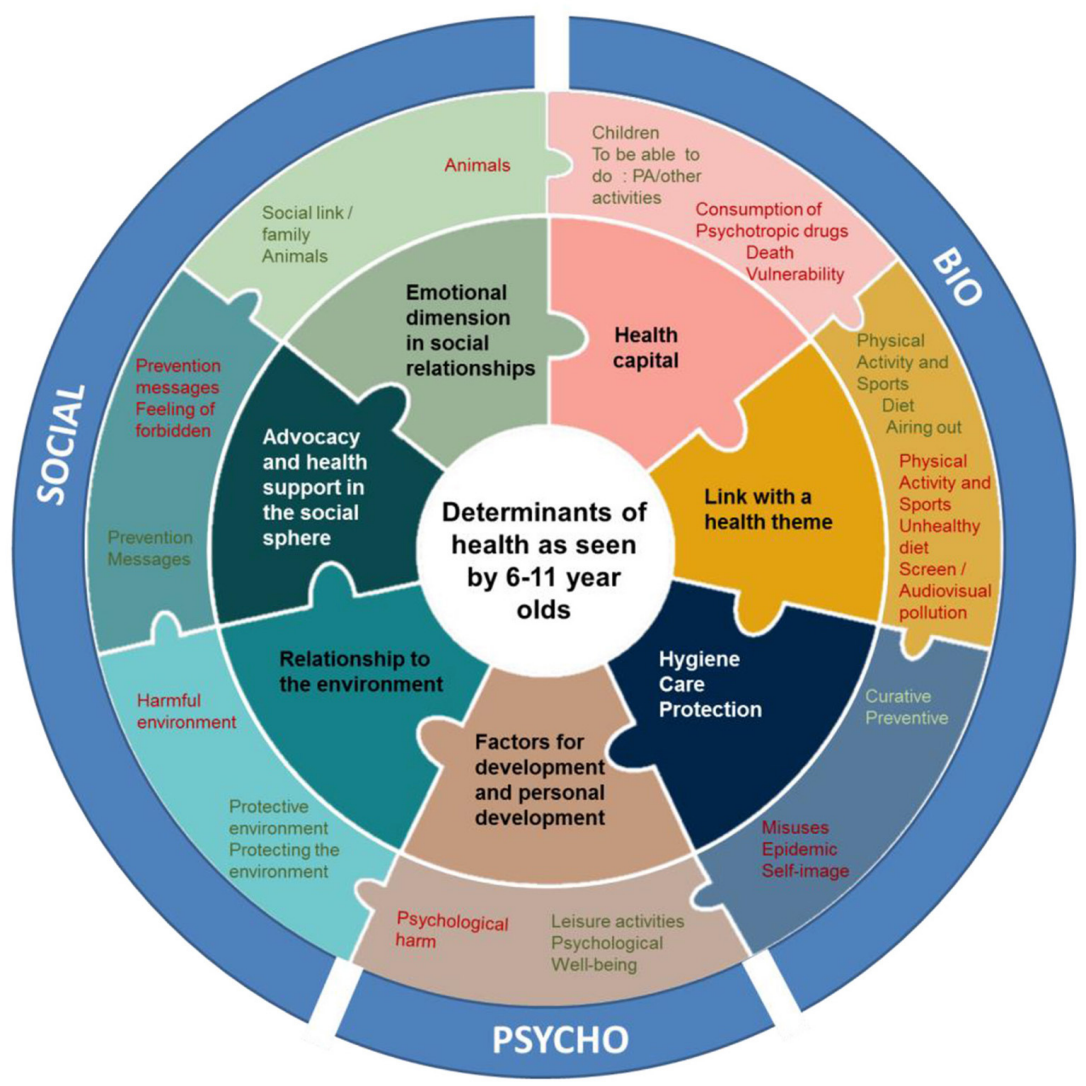
Modeling of the determinants of health expressed and perceived by 381 children aged 6–11 (6). PA, Physical activity.

The second added value lies in the variety of results collected from the age of 6 and over the entire age group investigated. It is structured according to seven domains: health capital, themes of health, hygiene and care, factors of personal fulfillment and development, relationship to the environment, advocacy and health support in the social sphere, emotional dimension in social relationships and these seven domains contain a total of 28 categories, source of a modeling (Figure 1). The inner circle in Figure 1 represents the seven domains. The middle circle represents the 28 categories corresponding to their area of color. The outer circle illustrates how this model fits into the biopsychosocial model.

These seven domains show that, collectively, the children who were interviewed in this study address all of the broad domains of determinants of health and cancer. The results, all combined (e.Photoexpression^©^ and Photonarration), show that all the children tackled the 7 domains, 1,978 times and the 28 categories underlying categories, 2,685 times. One child was able to name several categories from the same field.

The methodology allowed this richness of expression of the children. On average 2.1 (±0.74) domains and 2.49 (±0.87) categories were addressed during the first phase of the survey (e.Photoexpression^©^). During the Photonarration 3.11 (±1.25) domains and 4.39 (±2.11) categories on average were mentioned. The use of these two tools, the e.Photoexpression^©^ and the Photonarration, in that order, allowed the children to densify their speech. For 5 out of 7 domains, Photonarration has enriched the e.Photoexpression^©^ (+21.6% on average):

- “Link with a health theme” (+32.7%);
- “Health capital” (+29.4%);
- “Hygiene/Care/Protection” (+26.6%);
- “Relation to the environment” (+13.4%);
- “Affective dimension in social relationships” (+5.7%).

The two domains enriched by the e.Photoexpression^©^ are “factors of personal fulfillment and development” (+6.5%) and “advocacy and support in the social sphere” (+4.5%). The contribution of the Photonarration concerning the domain “link with a health theme” is largely influenced by two categories: “healthy food” (+45.5%) and “unhealthy food” (+39%). The “health capital” area is strongly influenced by the “consumption of psychotropic drugs” category (+34.8%). This increase was measured by calculating the difference between the percentage of the domain addressed by the children in the Photonarration and the percentage of this same domain cited by the children in the e.Photoexpression^©^ and vice-versa.

For 18 categories out of 28 (64.3%), Photonarration enriches the e.Photoexpression^©^ by +11.8% on average. The e.Photoexpression^©^ increases the photonarration by +2.3% for 10 categories. We therefore observe a densification of the discourse between the two survey phases, the children referring to more domains during the Photonarration. When they address only one area for the e.Photoexpression^©^: “I chose this image because vegetables are good for you and also because it makes you grow taller” (AYCE1R1F); “Because smoking can kill” (AMCP12M), the identification of several domains at the same time is revealed in the Photonarration with denser and more complete productions: “flowers are good for health: bees go in, make honey and honey it protects against cancer” (AYCP2M); “It's not good to see wine, beer you can get cancer; if we catch fish there will be no more in the ocean; it pollutes cars; I made a drawing which represents a person who puts gasoline and it is not good to put gasoline otherwise it pollutes the planet, it puts bad air outside and we can catch cancer” (GUCE28F).

**Figure 2:**
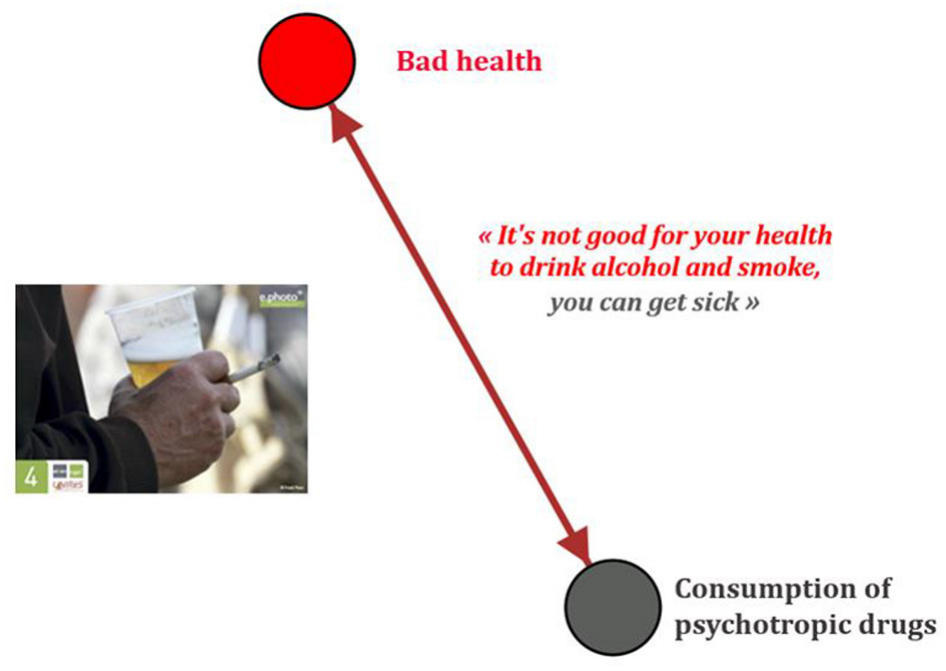
pictorial representation of simple conception systems e.Photoexpression^©^ “bad health” GEPHI^©^ (GUCE19F).

**Figure 3:**
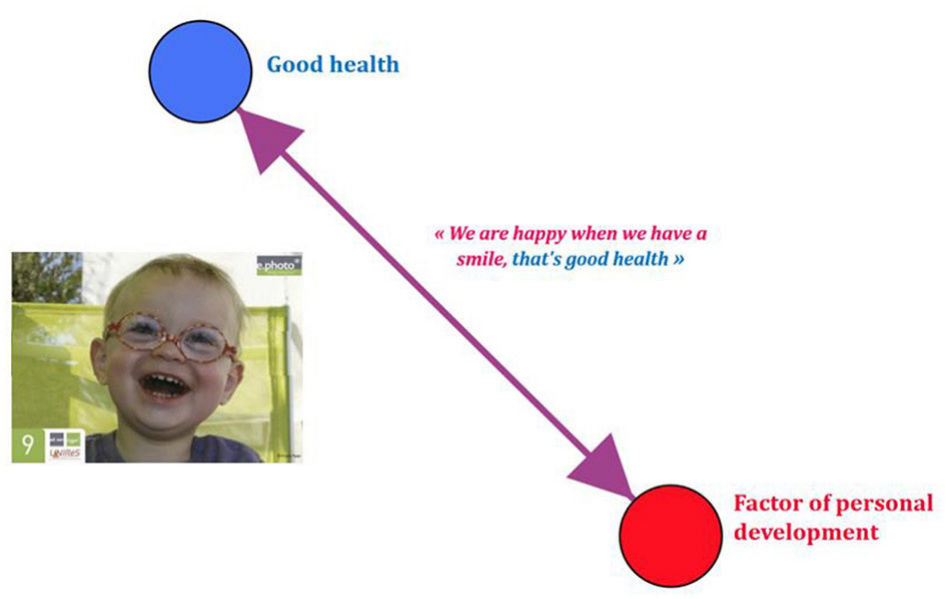
Pictorial representation of simple conception systems e.Photoexpression^©^ “good health” Gephi 9.2^©^ (GUCE19F).

The qualitative and quantitative analysis also highlights a major result: the density of the collection despite the age of the children. Whatever the field approached, the discourse is very rich among the youngest (Figures 2, 3). Children of 6 years (CP), 7 years (CE1), and 8 years (CE2) develop their words as much, if not more sometimes, as those of 9, 10 or 11 years (CM1 and CM2). It is interesting to value this result because many teachers volunteering for our study had prior doubts about the content of the productions of the youngest. They feared that the data lacked density and quality and therefore could not be used. This result shows that there is no new ground, that from an early age children have a lot to tell us and that they take us to horizons we would not have thought of.

### Discourse Tendencies

Analysis of these data highlights a third added value: that of two major trends among children in their conceptions of health and cancer determinants: (1) the under-representation of environmental determinants (20.48%) and (2) overrepresentation of individual determinants (76.49%). This result is in itself of great importance since it has so far never been stated (22). The environmental determinants include the domain “relationship to the environment.” The categories of the “relationship to the environment” domain include factors unfavorable to health (pollution, feeling of insecurity, precariousness, poor economic situation) and factors favorably influencing health (protection of the environment, good economic situation). The individual determinants refer to the domains “link with a health theme,” “health capital,” “factors for personal growth and development”; “emotional aspect in social relationships” and “hygiene, care and protection.”

These individual determinants reflect the discourse held by those around them and the prevention messages disseminated by the media, and exhaustively represent the modifiable risk factors for cancer (32). The parameters of accessibility to health services, social assistance, proximity services, education, housing, workplace and childcare are hidden by the children (3.03%). The verbatims that mention them are grouped under the domain “advice and support in the social sphere” through the categories “prevention messages” and “feeling of being forbidden.” The combined analysis of verbatims from e.Photoexpression^©^ and Photonarration makes it possible to stabilize these discourse tendencies. Children are essentially self-centered on personal behaviors, aspects of their daily life and the events that closely affect them, providing immediate leverage for action (6). According to them, the individual determinants have a predominantly favorable influence on health. Only the “health capital” domain gives a negative and anxiety-provoking input to the determinants of health (6). This is explained by the presence of categories related to this area, very negative such as death and consumption of psychotropic drugs (alcohol and tobacco). Here too, the combined analysis of the two survey phases makes it possible to stabilize and strengthen this result. As the verbatims linked to the photographs taken from the e.Photoexpression^©^ could suggest, the images present in the Photonarration show that the unfavorable factors take precedence over the environmental factors having a beneficial influence on health, making this area anxious from the point of view of children. The latter feel concerned by the environmental pollution linked to human activities (cars, waste) as well as by the importance of having a remunerative and fulfilling job in the future but do not address the theme of the risks linked to the environment by occupational exposure. They remain mainly focused on micro representations, touching an environment very close to their daily life and obscuring the overall context of society such as the economic situation of a country, its political context and the conditions of the labor market. The parameters of accessibility to health services, social assistance, local services, education, housing, place of work, childcare are very little mentioned (6, 31, 33). The few verbatims that mention it are grouped under the area “advocacy and support in the social sphere” through the categories “prevention messages” and “forbidden feeling.” This result emerges mainly from the Photonarration which shows the advantage of having this complementary phase to the e.Photoexpression^©^ to open up to other conceptions thus revealing a new trend. Highlighting the under-representation of the themes of accessibility to education, social and health services as well as that of the global, political, economic and cultural context and the over-representation of individual determinants that refer to personal responsibility, even guilt of a population in its capacity and its will to be in good or in bad health. Indeed, children stress the importance of a healthy and balanced diet as well as the importance of regular sports practice, but do not mention access to food and physical activity.

### Discourse Complementarity

A fourth added value of this multiphase protocol is to highlight a certain complementarity in the words of the children as the survey phases progress. It then becomes possible to describe how the conceptions expressed form a system among themselves, how they are articulated and with what force they are or are not linked in the words collected. The contribution of e.Photoexpression and Photonarration from a quantitative and qualitative point of view guarantees for each child a continuity in the discourse which allows to deepen his or her conceptions and to know how he or she creates or not the meaning between the determinants of the health and cancer. We know that, for these age groups, this is a real methodological issue, especially when we are interested in the perception of the determinants. This protocol shows that children have a much broader perception than one might ascribe to them intuitively.

From a qualitative and quantitative point of view, the discourse intensifies and expands allowing a better understanding of how conceptions make a system, i.e., how they combine, how they interconnect and influence each other. During the e.Phoexpression, 98 children out of 381 linked determinants contributing to good health and 72 children linked factors having an unfavorable impact on health. During the Photonarration, 137 children out of 368 made a link between at least two determinants favorable to health, protective of cancer, and 115 children linked together determinants unfavorable to health that could lead to cancer. This analysis was carried out at two scales: collective and individual. Looking at conceptions at the individual level shows that some go so far as to link four to five determinants together to talk about health or cancer. This also reveals the value of multiple collection phases to characterize conceptions systems. Take the example of the conceptions used by a child in CE1 during the two collection phases to talk about the determinants of health and cancer. During the first phase around e.Photoexpression^©^, the child does not establish a link between different determinants of health (Figures 2, 3). To express poor health, her choice fell on the photograph of someone drinking alcohol and smoking a cigarette, stating that “it is not good for your health to drink alcohol and to smoke, you can be sick.” Below, his or her conception of ill health is pictorially represented using Gephi 9.2^©^ software:

To describe good health, he or she chose a photograph representing a child who smiles because “we are happy when we have a smile, that's good health.” His or her conception of good health is represented below, pictorially, by the Gephi 9.2^©^ software.

This child does not link two or more determinants. His or her conception of good and bad health is reflected in the identification of a single determinant because children were allowed to choose only one photograph. For many of them, a photograph represents only one conception. If children did not have this restriction, they could have chosen several photographs representing different conceptions. However, they chose the picture that (unconsciously) represented their most important conception of good or bad health. This justifies the interest of having an additional survey phase allowing them to choose several images to express themselves.

**Figure 4:**
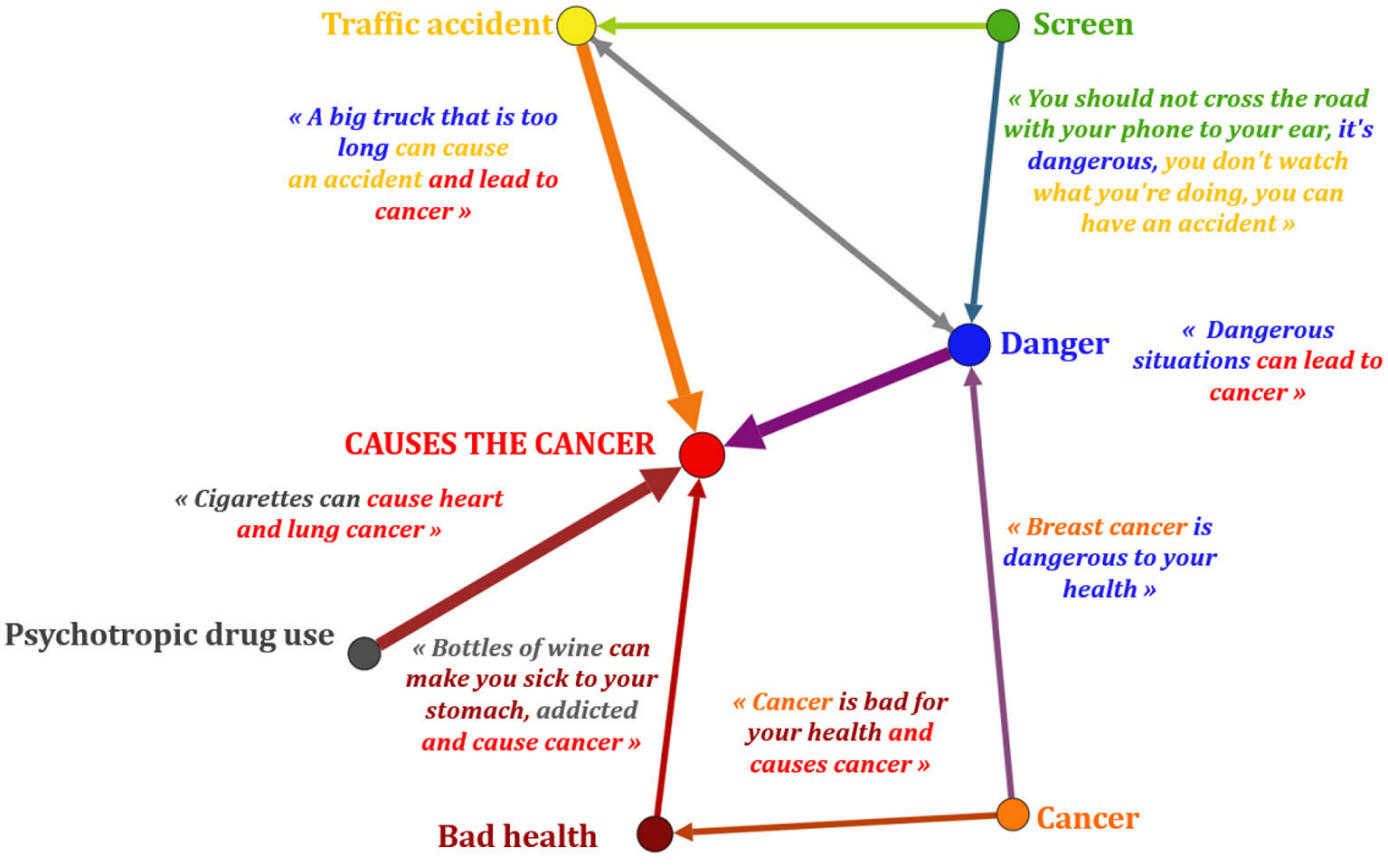
Pictorial representation of complex conceptions systems Photonarration ≪ causes cancer ≫ Photonarration - GEPHI 9.2^©^ (GUCE19F). The conceptions reveal more or less important links depending on the thickness of the line between different determinants unfavorable to health. Example: “screen”; “danger”; “accident”: these determinants are associated with each other, the child emphasizes their link and their causal effect: using their phone while crossing can be dangerous and can cause an accident.

**Figure 5:**
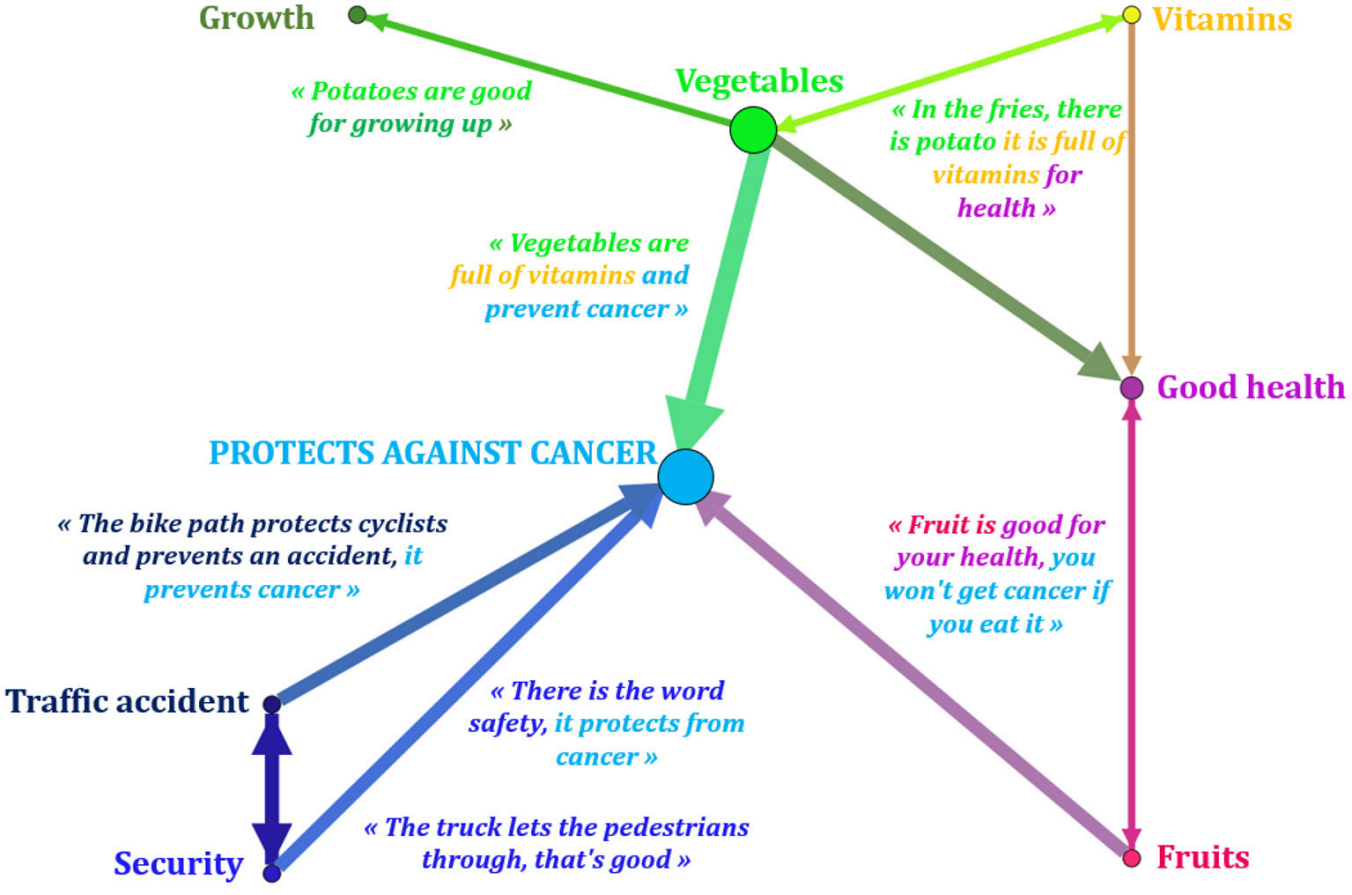
Pictorial representation of complex conceptions systems Photonarration ≪ protects against cancer ≫ - GEPHI 9.2^©^ (GUCE19F). The conceptions reveal more or less important links depending on the thickness of the line between different determinants favorable to health. Example: “vegetables”; “Vitamins”; “Growth”: these determinants are associated with each other, the child highlights their link and their causal effect: vegetables are full of vitamins, help to grow taller and protect against cancer.

During the second phase around Photonarration, he or she establishes a link between several determinants to describe what he or she believes leads to cancer and what, on the contrary, protects against disease (Figures 4, 5). These links were identified by a qualitative analysis and represented pictorially using the Gephi 9.2^©^ software:

We note that with the exception of screens, all of the categories represented are correlated with cancer. The consumption of psychotropic drugs is linked to cancer on two occasions: “cigarettes can cause heart and lung cancer”; “bottles of wine can make you sick to your stomach, addicted and lead to cancer” (GUCE19F). For this child, having cancer is “bad for your health and causes to cancer” (GUCE19F). He or she suggests that a recurrence is possible after a first cancer. He or she has twice addressed the question of the road accident linked to dangerous situations which may cause cancer: “a bick truck that is too long can cause an accident and lead to cancer”; “You should not cross the road with your phone to your ear, it's dangerous you don't watch what you're doing, you can have an accident” (GUCE19F). These ideas are predominant in this child since their links with cancer are more powerful than for the other determinants: the arrows connecting them to the central circle are thicker than the others.

There are two types of conceptions systems here: simple and complex. The words of the same child can reflect both a simple and a complex system of conceptions. The simple conceptions system represents the combination of only two determinants: “bottles of wine (determinant 1) can make you sick to your stomach, addicted (determinant 2) and lead to cancer.” The complex conceptions system shows the link between several determinants: “You should not cross the road with your phone to your ear (determinant 1), it's dangerous (determinant 2) you don't watch what you're doing, you can have an accident (determinant 3).” This distinction is also found in the favorable conceptions of this child through the determinants which, according to him, allow protection from cancer.

All the determinants represented here have a positive influence on health according to this child who considers that they protect against cancer. Fruits are “good for your health and it prevents you from getting cancer.” Road safety helps prevent accidents and watch out for pedestrians. For this child, security “protects against cancer.” (GUCE19F). Its conceptions individually constitute a simple conceptions system.

In another statement by this child, the presence of three interconnected determinants also reflects a complex conception system: “French fries contain potatoes, which are full of vitamins (healthy food - determinant 1) for health”; “Vegetables are full of vitamins, which prevent cancer (preventive - determinant 2).” He or she adds that “potatoes are good for growing (growth - determinant 3).” (GUCE19F). This child therefore considers that food plays a role in enriching health capital and contributes to reducing risks.

With these visual presentation strategies, it is then possible to quickly understand the complexity of the relationships that this child establishes between his or her conceptions: they are not independent of each other.

## Discussion

The e.Photoexpression^©^ and the Photonarration made it possible to collect 1,498 qualitative productions, produced by 381 children aged 6–11 years. The density and variety of the comments collected reinforce the validity and relevance of combining e.Photoexpression^©^ and Photonarration. They gave rise to the construction of a model of the determinants of health perceived by the children interviewed (Figure 1). The results of the “QC” (Question/Knowledge) phase of the Determ'Ados protocol, presented in another publication (34) are also numerous and highlight the children's desire to obtain more knowledge on the subject health and cancer (741 questions). We observe that despite a high number of statements (523 knowledge), the majority of them remain vague and approximate (34). The analysis of the results obtained during the e.Photoexpression^©^ and Photonarration phases makes it possible to identify how they mobilize this knowledge but also to better understand the way in which they perceive the factors influencing their health for good or bad and how they associate them. This analysis reveals two main trends. Their conceptions of the determinants of health and cancer refer to rationalities centered on the individual determinants (76.49%), minimizing the environmental determinants (20.48%). This is partly linked to the social context of stigmatization of individual behaviors aimed at promoting favorable or protective behaviors. In the discourse of children, therefore, they sometimes refer to the guilty effect of the recommendations and messages of prevention, to the personal responsibility of each one, that is to say to the capacity and the will to be in good or bad health (6). This multiphase protocol also highlights a certain complementarity in the children's comments throughout the survey phases. It then becomes possible to describe how the conceptions expressed form a system among themselves, how they are articulated and with what force they are or are not linked in the words collected.

Also, the methodologies used offer an ethical framework promoting, through image mediation, the necessary distance between the topic addressed and the children. The results prove it: on the children's side, no fear or stress was mentioned. It's a topic they can easily talk about and about which they have a lot to say. Image mediation creates a liberating space for words while respecting essential ethical considerations. It gives children the opportunity to structure their thinking and highlight the quality of their words. They find, through the picture, their own way of talking about health and cancer, in their own words. This method turns out to be particularly advantageous for the most fragile or those who find themselves in difficulty. Not imposing a framework and limit, the child is free to orient his or her thought and his or her production as he or she wishes, according to his or her own vision of the world but also with what he or she knows and what he or she has in his or her possession to formulate a response. Children in precarious or fragile situations are potentially put in a more favorable situation for them since they can understand the world as a whole. And it is this uniqueness and authenticity that makes up the wealth of the data available. Finally, the power of the image also lies in its ability to bring out unconscious ideas in the child. Indeed, he or she does not necessarily always have access to his or her own conceptions, sometimes retained in the unconscious (15). The mechanisms of the unconscious indeed control our decision-making, our behavior, our emotions, consciousness reflecting only part of the cognitive processes. The image is meaningful since it refers the child to his or her memories, to specific circumstances or situations buried in his or her unconscious, which he or she instinctively re-mobilizes to answer the question asked. Image mediation tools allow you to stay off-center, avoiding talking about yourself, while remaining on the topic being addressed. Indeed, the child “leads” his or her speech from the photographs. He or she then becomes an actor in the survey and provides a personal interpretation of the photographs he or she has chosen (35). Making this choice is a pleasant and motivating activity (36), quick to implement and enjoyable. Children tend to take a strong interest in any form of pictorial representation, especially photographs (37). They can provide a clear picture of the point of view expressed, which can then be explored with it in more depth (38), these visual aids being suitable for further discussion (39). They strengthen the building of a relationship of trust between researchers and participants.

One of the limitations that can be perceived concerns the data analysis and, more specifically, the researcher's interpretation of children's wording. In this study, the protocol in place prevents this bias. The data were subjected to a triple encoding procedure described above in the methodology in order to avoid over-interpretation. Moreover, the approach taken is based on a detailed and accurate content analysis that respects the children's words and remains faithful to their discourse. The other limitation that can be identified is that we are dealing with two themes that are apparently distinct. However, it was demonstrated in the introduction of this article that the “determinants of health” and the “determinants of cancer” are similar (4). What influences our health can also explain what can lead or protect us from cancer (5). These concepts are close and can therefore be analyzed in the same way.

Moreover, the passage between the two collection phases is a time for learning and acquiring concepts which contributes to the development and the progression of children's thinking on the theme in question.

The e.Photoexpression^©^ provides 40 images with a choice of only two images. The approach based on the theme of global health and the limited choice of images (good health and bad health) made it possible to limit the first phase of the survey. Indeed, this precise instruction gave each child room to express themselves. Composed of photographs representing the biopsychosocial register (drugs, a smile, the family…) but also more neutral photographs (a flower, a landscape…), the e.Photoexpression^©^ leaves freedom in what it gives to see, offering a very wide range of children's conceptions. It helps to structure thinking and guarantees a step-by-step progression, while respecting the cognitive process of adaptation and acceptance of the child's thinking (40). This tool promotes a first markup and sets a large framework, necessary to introduce the subject and let the children enter gradually and at their own pace in the process. This first phase gives them confidence, allows them to confide and thus legitimizes the second phase of the survey. Photonarration, which leaves complete flexibility in the number of images to choose from among fifty magazines, is in itself a source of uncertainty and instability. The gradual methodological process with a first markup by the e.Photoexpression^©^ made it possible to secure the child and make him capable of managing his or her fear of giving the “right” or “bad” answer and thus giving free rein to the expression of his or her conceptions of the determinants of cancer.

Photonarration, through its large panel of images, allows a free choice of expression and mobilization of conceptions. It gives children more possibilities for expression since there is no limit to the number of images to choose from in magazines. The narrative dimensions of this Photonarration promote a better understanding of the process by which the child connects conceptions relating to different domains (sport, diet, family, interpersonal relationships, emotions, feelings, etc.). It aims to overcome a recurring obstacle of the use of single photography which most often allows one to express only one healthy conception. It is a question, thanks to Photonarration, of moving toward a densification of the discourse and the emergence of an argument allowing to better understand how the conceptions form systems among themselves and to identify the emerging rationalities of this argumentation. It is therefore a deepening step that completes the first contact. It offers more freedom for the child to express himself on the subject. Nevertheless, the results of this research show that this evolutionary process is only possible through the presence of e.Photoexpression^©^ without which Photonarration would not be so rich.

The contribution of the multiphase lies in the densification of the discourse which intensifies and widens and allows a better understanding of the way in which the conceptions make a system, that is to say how they combine, how they interconnect. and influence each other. If the child mobilizes them in various situations, these conceptions also respond to a dynamic of interrelationships. He or she can therefore refer to a healthy conception allowing him to analyze a situation and use one or more others to find points of stability or solutions to this same situation. This is constitutive of a rationality specific to each child. This study therefore reveals a progression of children's conceptions and especially of their conception systems. For the e.Photoexpression^©^, only one determinant is cited to answer each of the questions asked. No link is therefore established. The Photonarration shows that there are many conceptions systems. Whether in a favorable or unfavorable approach, the child establishes connections between two or even three or four determinants which revealed that there are two levels of system of different conceptions: simple system and complex system. The discourse becomes denser and we are witnessing the emergence of an argument linked, among other things, to our progressive and complementary methodology. Multiphasing constitutes the innovative aspect of the tools used: the e.Photoexpression^©^ and Photonarration are complementary and inseparable to bring out concepts on health and cancer.

By this demonstration, we can confirm that photography is a powerful tool of collection (gives much to see quickly) whose relevance increases by diversifying its uses. All these elements of methodological discussion raise other points of questioning and new perspectives. Following this logic of diversification, we could consider establishing additional phases of collection from other tools mobilizing photography in order to enrich our corpus. It would then be interesting to imagine a new collection modality built around the “Photoelicitation” tool which, like e.Photoexpression^©^ and Photonarration, is based on images. An analysis of the international literature seems to show that Photoelicitation is an interesting tool for investigating health conceptions and conception systems (41). This new phase would complement and enrich the data collected through e.Photoexpression^©^ and Photonarration with another approach and a different use of photography. It is not a selection from a corpus, but a photograph to be taken by the child his or herself, a bias, a different commitment that amplifies the place and action of the child in the research process.

Our investigative methodology in two successive phases has therefore enabled us to reveal that children, from an early age, have a wide range of conceptions of the determinants of health. The range of conceptions indicates the effectiveness of the method. By adapting to the children's level of writing (dictation to adults), these collection tools allow a heterogeneous panel of children (age and social context) to express themselves freely, to verbalize and to mobilize conceptions which will be revealed or not and in a more or less distinct way. They have their own conceptions and manage to structure their thinking in an explanatory logic that it is essential to take into account. These different approaches offer new content and are above all complementary. They create a funnel effect, starting from the global and moving toward the specific, thus forming a reassuring cognitive path for the child. A generalist entry on health then oriented toward cancer favors a progressive approach. The added value of the multiphase lies in this process, this gradual methodological operation which facilitates the accompaniment of the child in a mental process of adaptation to the subject.

These research results, transferable into interventions and current practices, will allow those in charge of prevention to act more effectively, closer to the conceptions and needs of children.

## Data Availability Statement

The original contributions presented in the study are included in the article/supplementary material, further inquiries can be directed to the corresponding author/s.

## Ethics Statement

Ethical advice from the CPP SUD-EST VI - Ref: 2019/CE 10.

## Author Contributions

DM made a substantial contribution to the data acquisition, interpretation and analysis as well as to the total writing of the manuscript. PF and GC participated in the data acquisition, interpretation and analysis. PF, GL, and GC were recognized at the critical review of the manuscript for intellectual reasons. BP's contribution mainly concerns data analysis, being a statistician by profession. PF has definitely approved the version to be released. All authors contributed to the article and approved the submitted version.

## Funding

This original research was funded by Ligue Nationale Contre le Cancer.

## Conflict of Interest

The authors declare that the research was conducted in the absence of any commercial or financial relationships that could be construed as a potential conflict of interest.

## Publisher's Note

All claims expressed in this article are solely those of the authors and do not necessarily represent those of their affiliated organizations, or those of the publisher, the editors and the reviewers. Any product that may be evaluated in this article, or claim that may be made by its manufacturer, is not guaranteed or endorsed by the publisher.

## Footnotes

^1^Pédagogie Interactive en Promotion de la Santé: Interactive Pedagogy in Health Promotion.

^2^Institut National de la Propriété Industrielle: National Institute of Industrial Property.

^3^Article 12.1 of the CRC - principle of democracy, article 13 - freedom of expression, article 36 - protection against exploitation and article 3.3 - competence of bodies responsible for the health and protection of human beings. children.
